# Chinese herbal medicine for dyslipidemia: protocol for a systematic review and meta-analysis

**DOI:** 10.1097/MD.0000000000013048

**Published:** 2018-11-02

**Authors:** Caihong He, Peng Fu, Kexin Zhang, Qing Xia, Yunmei Yang, Liangzhen Xie

**Affiliations:** a First Affiliated Hospital, College of Medicine, Zhejiang University, Hangzhou; b First Affiliated Hospital, Heilongjiang University of Chinese Medicine, Harbin, China.

**Keywords:** Chinese herbal medicine, dyslipidemia, protocol, systematic review

## Abstract

**Background:**

Dyslipidemia is commonly characterized by the abnormal quantity and quality of lipids in plasma, which is strongly associated with an increased risk of cardiovascular disease and also a major cause of morbidity and even leads to mortality. In China and East Asia, Chinese herbal medicine has been widely used to treat diverse diseases for thousands of years. As an important means of traditional Chinese medicine treatment, Chinese herbal medicine plays a more important role in the treatment of dyslipidemia. The aim of this study is to assess the efficacy and safety of Chinese herbal medicine for dyslipidemia.

**Methods:**

Seven electronic databases (included The Cochrane Library, MEDLINE, Embase, CNKI, VIP, CBM, and WANGFANG) will be searched regardless of publication date or language. Randomized controlled trials will be included if they recruited participants with dyslipidemia for assessing the effect of Chinese herbal medicine vs control (placebo, no treatment, and other therapeutic agents). Primary outcomes will include serum lipid and advent events. Two authors will independently scan the articles searched, extract the data from articles included, and assess the risk of bias by Cochrane tool of risk of bias. Disagreements will be resolved by discussion among authors. All analysis will be performed based on the Cochrane Handbook for Systematic Reviews of Interventions. Dichotomous variables will be reported as risk ratio or odds ratio with 95% confidence intervals (CIs) and continuous variables will be summarized as mean difference or standard mean difference with 95% CIs.

**Results:**

This review will be to assess the efficacy and safety of Chinese herbal medicine for dyslipidemia.

**Conclusions:**

Our findings will assist clinicians and health professionals make clinical decisions regarding dyslipidemia prevention, and promising way for prevention and treatment of patients with dyslipidemia.

**Ethics and dissemination:**

This study is a protocol for systematic review of Chinese herbal medicine as a treatment of dyslipidemia. This review will be published in a journal and disseminated in print by peer-review.

**Systematic review registration:**

PROSPERO (CRD42018085556).

## Abstract 2

Supplemental Digital Content is available in the text

## 1 Introduction

### 1.1 Disease burden of dyslipidemia

Dyslipidemia, defined as the abnormal quantity and quality of lipids in plasma, is strongly associated with an increased risk of cardiovascular disease (CVD) and also a major cause of morbidity and leads to mortality. Dyslipidemia is commonly characterized by low levels of high-density lipoprotein cholesterol (HDL-C) and high levels of triglyceride (TG), total cholesterol (TC), and low-density lipoprotein cholesterol (LDL-C).^[1,2]^ Millions of people around the world are affected dyslipidemia.^[3]^ It is estimated that prevalent cases of dyslipidemia in the 9 major countries will increase at the rate of 1.76% per year to surpass 500 million in 2022.^[4]^ In China, with rapid economic growth and associated lifestyle changes, the level of dyslipidemia has gradually increased for nearly 30 years, the prevalence of dyslipidemia also increased significantly. According to a 2012 survey, the overall prevalence of dyslipidemia in Chinese adult is 40.40%, which has risen sharply compared to the result from 2002.^[5]^ Recent data show that the prevalence of dyslipidemia levels in some areas has increased further,^[6–9]^ unfavorable trends in lipid levels have occurred among adults.

Paradoxically, with the update of the guidelines for the control of dyslipidemia, more effective interventions and drugs were introduced and applied to clinic; however, the number of people with dyslipidemia has no reduction. Dyslipidemia abnormalities are still a very serious burden of disease affecting human health, more effort and effective strategies are required for the prevention.

### 1.2 Advantages of Chinese herbal medicine for the treatment of dyslipidemia

In clinical practice, a variety of therapeutic strategies for dyslipidemia were used, involved pharmacotherapy, lifestyle modification, liposuction and plasma exchange, etc. Stains are widely used as the 1st line drug for dyslipidemia, especially hypercholesterolemia, which is likely to achieve a reduction of TG and LDL-C. Although it is generally well-tolerated in most of patients with dyslipidemia, each lowering-lipid drug has certain side effects, such as statin may result in myalgias and muscle weakness, reduced energy, increased fatigue, liver enzyme elevations, worsening hyperglycemia, and risk of incident diabetes.^[10]^ Along with the long-term treatment with statins in combination with other hypolipidemic drugs or alone, its safety and the medication compliance of patient had attracted a particular attention in clinic.^[11]^ So more and more comprehensive strategy is required in clinical practice. Chinese herbal medicine is one of the most common treatment in traditional Chinese medicine (TCM), using the medicine derived from botanical sources to treat medical conditions. In China and East Asia, Chinese herbal medicine has been widely used to treat diverse diseases for thousands of years, and have been involved in dyslipidemia intervention for decades, which enriched the treatment of dyslipidemia greatly. The distinction between humans and experimental animals is that the human population is a total mixture, unlike selected batches of laboratory animals (same age, weight, sex, strain, etc).^[9]^ Dyslipidemia is also the result of multiple factors, including intrinsic and extrinsic causes, such as age, diabetes, hypertension, body size, smoking status, family history, alcohol consumption, etc. The clinical manifestations of dyslipidemia are also complex, causing a variety of disorders, not only in terms of elevated serum lipid. For this reason, we need to take a holistic approach to different individuals and different qualities. This is fully consistent with the characteristics of TCM syndrome differentiation. As an important means of TCM treatment, Chinese herbal medicine plays a more important role in the treatment of dyslipidemia.

### 1.3 Reasons for doing the study

With the knowledge and accumulated practice passing from generation to generation, many herbs have been proven to have therapeutic effects on dyslipidemia, such as Radix Et. Rhizoma Rhei (Da Huang), *Polygonum cuspidatum* (Hu Zhang), Semen Cassia (Jue Ming Zi), Rhizoma Coptidis (Huang Lian), *Scutellaria baicalensis* (Huang Qin), *Gynostemma pentaphylla* (Jiao Gu Lan), Radix Puerariae (Ge Gen), Fructus Crataegi (Shan Zha), Fermentum Rubrum (Red Yeast Rice), Rhizoma Chuanxiong (Chuan Xiong), Radix Salviae Miltiorrhizae (Dan Shen), Rhizoma Curcumae Longae (Jiang Huang), Rhizoma Alismatis (Ze Xie), Semen Plantaginis (Che Qian Zi), Folium Nelumbinis (He Ye), Radix Astragali (Huang Qi), Radix Ginseng (Ren Shen), and Radix Polygoni Multiflori (He Shou Wu).^[11–15]^ A total of 57 TCM formulas, including Long-Dan-Xie-Gan Tang, Wen-Dan Tang, Er-Chen-Tang and Xue-Fu-Zhu-Yu Tang, Wei-Ling TangYi-Guan-Jian and Qi-Ju-Di-Huang Wan, You-Gui-Wan and Shen-Ling-Bai-Shu San, etc, have been approved by the China Food and Drug Administration to treat hyperlipidemia.^[13,14]^ Recently, many studies also demonstrated a favorable effect of Chinese herbal medicine for treating dyslipidemia.^[16–19]^ However, no comprehensive evaluation have been reported in recent years. So we aim to gather the up-to-date information on Chinese herbal medicine for dyslipidemia and evaluate the potential benefits and harms of the use of Chinese herbal medicine, and move forward to help inform clinical decisions.

## 2 Methods and analysis

### 2.1 Objectives and registration

This review will be to assess the efficacy and safety of Chinese herbal medicine for dyslipidemia. This review protocol is registered in the International Prospective Register of Systematic Reviews (PROSPERO: CRD42018085556). Additionally, this review will adhere to the Preferred Reporting Items for Systematic Reviews and Meta-Analyses Statement.^[20]^

### 2.2 Eligibility criteria

#### 2.2.1 Types of studies

Randomized controlled trials (RCTs) will be included in this systematic review regardless of publication status and language. Quasi-randomized controlled trials (QRCTs) and nonrandomized studies will be excluded.

#### 2.2.2 Types of participants

Participants with primary dyslipidemia, who were 18 years or older, were included regardless of their age, sex, or race if the diagnosis of dyslipidemia was made by “Screening and management of lipids” or any other reasonable criteria. Dyslipidemia was defined as high TC, high TGs, low high-density lipoprotein, and elevated LDL-C. Secondary dyslipidemia was excluded, which included diabetes, hypothyroidism, nephrotic syndrome, liver and gallbladder disease, and so on.

#### 2.2.3 Patient and public involvement

In this study, there is no patient and public involvement in consideration of this protocol for a systematic review.

#### 2.2.4 Types of interventions

All types of Chinese herbal medicine will be included. There is no limitation on the number of herbs, administration methods, dosage, or duration of treatment. The comparisons will be either with other therapeutic agents, such as statin and fibrate, or with no other treatment or placebo.

### 2.3 Types of outcome measures

The main outcome measures we sought at the end of treatment and at maximal follow-up after completion of the treatment were as follows.

#### 2.3.1 Primary outcomes

1. Serum lipid levels (including TC, TG, LDL-C, and HDL-C)
2. Adverse events

#### 2.3.2 Secondary outcomes

1. Health-related quality of life
2. Weight, body mass index, waist circumference, waist-to-hip ratio (WHR)

### 2.4 Search methods for the identification of studies

#### 2.4.1 Electronic searches

We will search the following electronic databases regardless of publication date or language:

1. The Cochrane Library
2. MEDLINE
3. Embase
4. Chinese BioMedical Database (CBM)
5. China National Knowledge Infrastructure (CNKI)
6. Chinese VIP Information (VIP)
7. Wangfang Database

#### 2.4.2 Other sources

We will scan the reference lists of reviews and retrieve articles for additional studies. In addition, we will search Chinese Clinical Trials Registry (ChiCTR) (http://www.chictr.org.cn/), ClinicalTrials.gov (https://clinicaltrials.gov), and Google scholar (http://scholar.google.com).

#### 2.4.3 Search strategy

We will model participant strategies for databases on the search MEDLINE and CNKI (see supplementary table). If this was the case, we would have modified electronic search strategies to incorporate these terms. We included studies published in any language.

### 2.5 Data collection and analysis

#### 2.5.1 Selection of studies

**Figure 1:**
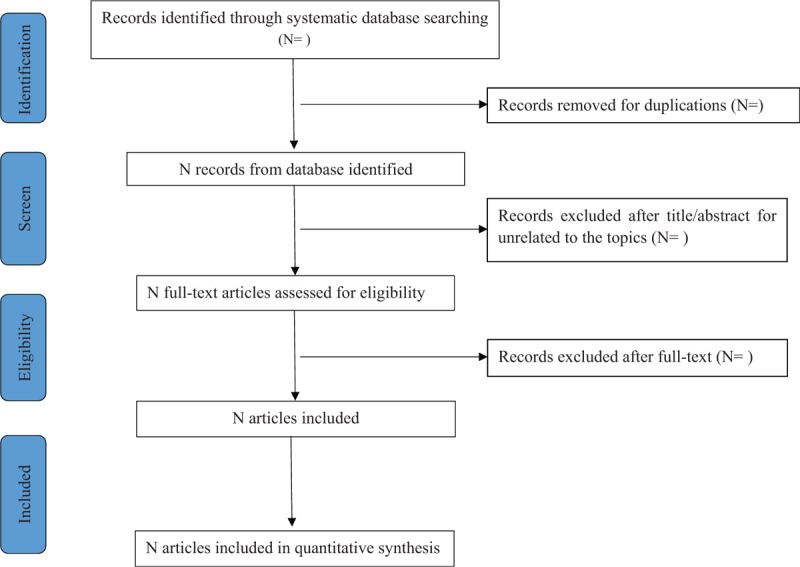
Flow chart of study selection.

To determine the studies to be searched further, 2 review authors (CHH and PF) will independently scan the titles and abstracts of all articles identified from electronic databases. Full-text articles will be scanned for all potentially relevant articles. If there is any disagreement on the selection of articles, they will be discussed with the 3rd author (YMY). The selection process will be shown in a Preferred Reporting Items for Systematic Review and Meta-analysis (PRISMA) flow chart (Fig. 1).

#### 2.5.2 Data extraction and management

For all studies included, 2 review authors (CHH and PF) will independently extract the relevant information using a standard data extraction table. Information will include publication of year, author, participants, intervention, control, duration of intervention, outcomes, and methodologic characteristics. Disagreements will be resolved by discussion by arbiter (LZX).

#### 2.5.3 Assessment of the risk of bias in the included studies

The risk of bias will be assessed independently by 2 authors (CHH and PF) using the Cochrane tool of risk of bias (V.5.1.0). The following items will be assessed: random sequence generation (selection bias), allocation concealment (selection bias), blinding (performance bias and detection bias), incomplete outcome data (attrition bias), selective outcome reporting (reporting bias), and other bias. The judgments of evaluated domains will include high, low, and unclear. Disagreements will be resolved by discussion by arbiter (LZX).

#### 2.5.4 Measurement of the treatment effect

Continuous variables will be reported as mean difference (MD) with 95% confidence intervals (CIs). For different measurement scales, standardized MD (SMD) analysis with 95% CI will be used, for example, TC and TG. Categorical variables will be summarized as risk ratios (RRs) or odds ratio (OR) with 95% CIs, for example, adverse event. All analysis will be performed based on the Cochrane Handbook for Systematic Reviews of Interventions.^[21]^

#### 2.5.5 Units of analysis issues

All parallel-designed studies will be included in this review. For cross-over trials, only the 1st treatment period data will be analyzed. For studies with multiple control groups, the unit of analysis will be used to each of all control groups.

#### 2.5.6 Dealing with missing data

For insufficient or missing data, we will contact the authors by e-mail or phone as much as possible. All analysis will be performed based on intent-to-treat principle.

#### 2.5.7 Assessment of heterogeneity

Heterogeneity will be identified by visual inspection of the forest and tested by standard Chi-squared statistic and a significance level of 0.1. Additionally, the *I*^2^ statistic will be used to examine heterogeneity for quantifying inconsistency in the included studies. Fixed or random effects models will be performed in meta-analysis. If *I*^2^ >0.5, random effects models will be used.^[21]^

#### 2.5.8 Assessment of reporting biases

Funnel plots will be used to assess the potential for small study bias if there are sufficient studies. Asymmetry of funnel plots will suggest possible small study effects and the results will be explained cautiously.^[22,23]^

#### 2.5.9 Data synthesis

If there are sufficient studies and comparable outcomes, we will perform a meta-analysis using random effect modeling.

1. Chinese herbal medicine vs conventional medical treatments
2. Chinese herbal medicine vs no treatment
3. Chinese herbal medicine vs placebo
4. Chinese herbal medicine plus conventional medical treatment vs conventional medical treatment only

#### 2.5.10 Subgroup analysis and investigation of heterogeneity

Subgroup analysis will be performed to explore the differences in the methodologic quality, subtypes of dyslipidemia, race/ethnicity, and types of herbal medicine.

#### 2.5.11 Sensitivity analysis

Sensitivity analysis will be performed to test the robustness of findings if there are sufficient studies included. The factors on effect are as follows:

1. Methodologic quality: analysis will be performed excluding studies of poor methodologic quality
2. Published status: analysis will be performed excluding unpublished studies
3. Sample size: analysis will be performed excluding small sample size studies
4. Diagnostic criteria: analysis will be performed in studies of the same diagnostic criteria
5. Race/ethnicity: analysis will be performed in studies of the same race or ethnicity

#### 2.5.12 Confidence in cumulative evidence

In this study, the level of evidence on outcomes will be assessed using an approach based on the Grading of Recommendations Assessment, Development and Evaluation (GRADE). The quality of the body of evidence will be assessed based on 5 factors, including study limitations, effect consistency, imprecision, indirectness, and publication bias. The assessments will be categorized as high, moderate, low, and very low quality.

## 3 Discussion

The effective treatment of dyslipidemia is significant. Chinese herbal medicine has an advantage in treating dyslipidemia,^[24]^ is able to help make up for the deficiency of current treatment, and is worth studying. The Cochrane Database of systematic reviews on dyslipidemia in 2011^[18]^ and 2013^[17]^ were focused on hypercholesterolemia and hypertriglyceridemia, respectively. All forms of dyslipidemia will be included in our review. We will summarize the available evidence for Chinese herbal medicine on dyslipidemia, and evaluate the effectiveness and the adverse effects of these treatments on dyslipidemia. Our findings may assist clinicians and health professionals make clinical decisions regarding dyslipidemia prevention, and promising way for prevention and treatment of patients with dyslipidemia. For the status of this study, we begin formal screening of search results against eligibility criteria.

### 3.1 Ethics and dissemination

Ethical approval is not required, in consideration of this protocol for a systematic review. In this study, there will be no participants recruited, and no data gathered from participants. This review will be disseminated by the approach of peer-reviewed publications.

## Author contributions

LZX, YMY, CHH, and PF developed the study protocol. LZX, YMY, CHH, and PF developed the search strategy. CHH and PF will scan the included studies, extract the data and assess the risk of bias. LZX and YMY will act as an arbiter if there is any disagreement in this study. CHH and PF will perform data analysis with supervision of LZX. All authors (CHH, PF, KXZ, QX, YMY, and LZX) will contribute to data interpretation. CHH, PF, YMY, and LZX drafted and revised the manuscript. All authors have read and approved the final version of the manuscript.

**Conceptualization:** Caihong He, Peng Fu, Yunmei Yang, Liangzhen Xie.

**Data curation:** Caihong He, Peng Fu.

**Formal analysis:** Caihong He, Peng Fu, Liangzhen Xie.

**Methodology:** Liangzhen Xie.

**Software:** Qing Xia, Liangzhen Xie.

**Supervision:** Yunmei Yang, Liangzhen Xie.

**Writing – original draft:** Caihong He, Peng Fu.

**Writing – review & editing:** Kexin Zhang, Qing Xia, Yunmei Yang, Liangzhen Xie.

## Supplementary Material

Supplemental Digital Content
