# Supplementary material for: Chinese herbal medicine for dyslipidemia: protocol for a systematic review and meta-analysis
Source: Medicine (Baltimore). 2018 Nov 2;97(44):e13048. doi: 10.1097/MD.0000000000013048 (PMC6221744; doi:10.1097/MD.0000000000013048)
Supplement: Supplemental Digital Content [file medi-97-e13048-s001.docx]

**Search strategies**

Detailed search strategies will be made. Additional keywords of relevance could have been detected during the search process and we would have modified electronic search strategies to incorporate these terms. We take MEDLINE and CNKI as an example.

| MEDLINE |
| --- |
| 1. exp Dyslipidemias / or exp Hyperlipidemias |
| 2. (hypertrigly?erid?emia* or Hypertriacylgly?erid?emia*).tw,ot. |
| 3. (hypercholesterol?emi* or hyperlipoprotein?emia).tw,ot. |
| 4. ((HDL or LDL or cholesterol) adj6 (elevat* or ascend* or ris* or increas* or improv*)).tw,ot. |
| 5. or/1-4 |
| 6. exp Drugs, Chinese Herbal/ |
| 7. exp Phytotherapy/ |
| 8. exp Herbal Medicine/ |
| 9. exp Plants, Medicinal/ |
| 10. exp Plant Extracts/ |
| 11. exp Medicine, Kampo/ |
| 12. exp Medicine, Chinese traditional/ |
| 13. (herbal adj6 (remed* or extract* or preparation* or mixture* or medic*)).tw,ot. |
| 14 (phyto adj6 (drug* or pharmaceutical* or therap* or treatment* or medici*)).tw,ot. |
| 15. (Chinese adj6 (herb* or plant* or medic* or drug* or formul* or prescri*)).tw,ot. |
| 16. (plant* adj6 (preparation* or extract* or medic*)).tw,ot. |
| 17. (Chinese adj6 traditional medic*).tw,ot. |
| 18. botanical extract*.tw,ot. |
| 19. or/6-18 |
| 20. randomized controlled trial.pt. |
| 21. controlled clinical trial.pt. |
| 22. randomi?ed.ab. |
| 23. randomly.ab. |
| 24. trial.ab. |
| 25. or/20-24 |
| 26. (animals not (animals and humans)).sh. |
| 27. 5 and 19 and 25 not 26 |
| CNKI |
| 1. Dyslipidemias or hyperlipidemias |
| 2. Low density lipoprotein ascend or LDL ascend or High density lipoprotein reduce or HDL reduce or cholesterol ascend or TC ascend or high cholesterol or hypercholesterolemia |
| 3. Triglyceride ascend or high triglyceride or hypertriglyceridaemia |
| 4. 1 or 2 or 3 |
| 5. Chinese herbal medicine or Chinese medicine or Chinese and western or plants or herbs |
| 6. 4 and 5 |
